# Extent and location of tumor infiltrating lymphocytes in microsatellite stable colon cancer predict outcome to adjuvant active specific immunotherapy

**DOI:** 10.1186/2051-1426-3-S2-P405

**Published:** 2015-11-04

**Authors:** Annelies Turksma, Veerle Coupe, Marc Shamier, Kevin Lam, Vincent de Weger, Jeroen Belien, Alfons van den Eertwegh, Gerrit A Meijer, Chris Meijer, Erik Hooijberg

**Affiliations:** 1 VUMC/CCA, Amsterdam, Netherlands; 2 VUMC, Amsterdam, Netherlands; 3 VU University medical center, Cancer Center Amsterdam, Amsterdam, Netherlands; 4 Netherlands Cancer Institute, Amsterdam, Netherlands

## Purpose

To determine the prognostic and predictive value of tumor infiltrating lymphocytes (TIL) in colon cancer in a cohort of patients who previously took part in a trial on adjuvant Active Specific Immunotherapy (ASI).

## Background information

Vermorken et al.[1] conducted a multicenter clinical trial on adjuvant ASI for colon cancer patients. A vaccine consisting of irradiated autologous tumor cells admixed with the adjuvant Bacillus Calmette-Guérin bacteria has been evaluated. In that study, a comparison was made between surgery alone and surgery followed by adjuvant ASI treatment. The recurrence free interval for patients with stage II tumors was significantly extended for patients treated with surgery plus ASI compared to surgery alone, but not for stage III patients. A follow up study by de Weger et al.[2] showed that patients with stage II microsatellite stable tumors (MSS) benefited most from ASI treatment. The data on patients with microsatellite instable tumors (MSI) were inconclusive.

## Current experimental design

Here we determined the number and location of CD3+ and CD8+ cells in archival tumor samples of 106 MSS colon cancers. Disease specific survival (DSS) and recurrence free interval (RFI) were evaluated in detail at the 5 year post-treatment time point. First, we investigated the prognostic value of stromal and intraepithelial T cell infiltrates independent of treatment arm. Second, we investigated the predictive value of stromal and intraepithelial T cell infiltrates for clinical outcome after ASI treatment. For the analysis we used continuous data on T cell counts comparing the treatment effect of adjuvant Active Specific Immunotherapy in patients with high TIL to the treatment effect in low TIL.

## Results

Based on the data presented we concluded that 1) high numbers of stromal CD3 T cells have positive prognostic value measured as DSS for patients with stage II MSS tumors, and 2) high numbers of epithelial CD8 positive T cells have positive prognostic value measured as RFI for the group of patients with stage II MSS tumors as well as for the whole group (stage II plus stage III together). Furthermore we concluded that high numbers of preexisting stromal CD3 positive T cells are of positive predictive value in adjuvant ASI treatment measured as DSS as well as RFI.

## Conclusion

ASI therapy contributes to an improved DSS and RFI in patients with MSS colon tumors harboring high numbers of preexisting stromal CD3+ TIL.

## References

[B1] Vermorken Lancet 1999 353 9150 345 350 9950438 10.1016/S0140-6736(98)07186-4

[B2] de Weger CCR 2011 18 3 882 889

